# MDM2-p53 Interactions in Human Hepatocellular Carcinoma: What Is the Role of Nutlins and New Therapeutic Options?

**DOI:** 10.3390/jcm7040064

**Published:** 2018-03-27

**Authors:** Samy A. Azer

**Affiliations:** The Chair of Curriculum Development and Research Unit, College of Medicine, King Saud University, Riyadh 11461, Saudi Arabia; azer2000@optusnet.com.au; Tel.: +966-11-4699178; Fax: +966-11-4699174

**Keywords:** p53, MDM2, Nutlin, hepatocellular carcinoma (HCC), molecular pathogenesis, mechanisms, therapeutic approaches

## Abstract

Human hepatocellular carcinoma (HCC) is the fifth most common cancer and is associated with poor prognosis worldwide. The molecular mechanisms underlying the pathogenesis of HCC have been an area of continuing interest, and recent studies using next generation sequencing (NGS) have revealed much regarding previously unsettled issues. Molecular studies using HCC samples have been mainly targeted with the aim to identify the fundamental mechanisms contributing to HCC and identify more effective treatments. In response to cellular stresses (e.g., DNA damage or oncogenes), activated p53 elicits appropriate responses that aim at DNA repair, genetic stability, cell cycle arrest, and the deletion of DNA-damaged cells. On the other hand, the murine double minute 2 (MDM2) oncogene protein is an important cellular antagonist of p53. MDM2 negatively regulates p53 activity through the induction of p53 protein degradation. However, current research has shown that the mechanisms underlying MDM2-p53 interactions are more complex than previously thought. Microarray data have added new insight into the transcription changes in HCC. Recently, Nutlin-3 has shown potency against p53-MDM2 binding and the enhancement of p53 stabilization as well as an increment of p53 cellular accumulation with potential therapeutic effects. This review outlines the molecular mechanisms involved in the p53-MDM2 pathways, the biological factors influencing these pathways, and their roles in the pathogenesis of HCC. It also discusses the action of Nutlin-3 treatment in inducing growth arrest in HCC and elaborates on future directions in research in this area. More research on the biology of p53-MDM2 interactions may offer a better understanding of these mechanisms and discover new biomarkers, sensitive prognostic indicators as well as new therapeutic interventions in HCC.

## 1. Introduction

Human hepatocellular carcinoma (HCC) is associated with poor prognosis worldwide. The presence of microvascular invasion and occult metastasis is a significant cause of poor outcome in HCC [1]. Common contributing risk factors for the development of HCC are hepatitis viral infections (hepatitis B and C), chronic alcohol consumption, aflatoxin B1 exposure, and non-alcoholic fatty liver disease [2]. The risk of HCC is low in healthy livers and early stages of chronic liver disease, but sharply increases with the development of liver cirrhosis. As not all patients with risk factors develop cancer, the genetic alterations in hepatocytes may play a significant role in malignant transformation, uncontrolled cellular proliferation, and metastasis. Molecular changes associated with HCC development include telomere shortening, mutations of the telomerase reverse transcriptase (TERT) gene [3], loss of the p53 checkpoint function [4], and the evolution of aneuploidy. In mice with telomere dysfunction, the impairment of tumor progression was associated with an activation of p53-dependent DNA-damage responses [3]. In another study that used mice with telomere dysfunction, the mutation of p53 alleviated tumor suppression and accelerated chromosomal instability and cancer [5]. p53 is the most altered gene in human cancer, and HCC is frequently associated with an inactive form of p53, which contributes to tumor progression and metastasis.

p53 is a tumor suppressor gene and functionally acts as “a major guardian of the genome”. It has several functions including cell cycle control, apoptosis, and maintenance of genomic stability. Mutations or deletions of p53 have been observed in nearly half of human cancers including HCC, indicating its possible role in HCC pathogenesis. Murine double minute 2 (MDM2) is a crucial regulator of the p53 pathway and has a negative effect on p53 function [6,7]. By binding to the p53 protein, MDM2 inhibits its transcription function and mediates its degradation via the ubiquitination system. In the meantime, p53 positively regulates MDM2 expression, which results in a negative auto-regulation feedback loop [8].

Despite advances in the treatment of some other cancers, there is no specific therapy for HCC, and currently, surgical resection and chemotherapy are the most commonly practiced management options. Therefore, understanding the pathogenesis of HCC and the molecular basis of its development could enable the discovery of new therapeutic options. This review aims at exploring the molecular mechanisms involved in the pathogenesis of HCC with particular emphasis on the MDM2-p53 pathways and the proposed role of MDM2 to repress p53 activity. By understanding the mechanism by which MDM2 is involved in the pathogenesis of HCC, we can identify possible tools to suppress HCC and possibly identify new therapeutic interventions. This review also explores the role of Nutlins as a promising therapeutic intervention, and in light of this understanding, we will discuss the role of molecular biology in the identification of new biomarkers of HCC, the use of MDM2-p53 interactions as prognostic indicators, and future therapeutic approaches in treating HCC.

## 2. Tumor Suppressor p53

The human p53 gene codes for a nuclear protein that plays a major role in cell replication. It spans about 20 kb of DNA. Using southern filter hybridization of DNAs from a human-rodent hybrid, it was localized to the short arm of chromosome 17 (17 p. 13.1) [9]. The gene is composed of 11 exons. Earlier research has shown that the human p53 protein consists of 393 amino acids and contains four major functional domains. Details on the biochemical structure of p53 gene and the p53 protein are discussed elsewhere in the literature e.g., [10].

The observation that Simian virus 40-transformed cells express new species of proteins that can be precipitated by the anti-simian virus 40 tumor serum revealed the identification of a protein with an apparent molecular mass of 53 kDa, which has been identified as the p53 protein [11]. Further studies revealed that treatment of Simian virus 40 (SV40)-infected monkey cells with fluorophenylalanine (FPA) caused an increase in cellular uptake of thymidine and progressive inhibition of both viral and cellular DNA synthesis [12]. Cellular studies have revealed that transformed mouse cells express p53, which include leukemias, spontaneously transformed fibroblasts, and murine sarcoma virus [13,14]. The wild-type p53 protein is found to form complexes with the hepatitis B virus X protein (HBX) and the virus sequence-specific DNA binding is inhibited in vitro. The wide range of changes of p53 functions made by HBX contributes to the development of human hepatocellular carcinoma [15]. The identification of p53 in transformed cell lines and human leukemia cells suggests that p53 may contribute to the phenotype of certain leukemias [16,17]. In addition to its role in controlling the transition of resting normal cells from the G0-state of the cell cycle to the S-phase, the expression of p53 protein appears to be necessary for the process of physiological cycling cells. The expression of p53 in growth-arrested normal cells is controlled at the transcriptional/post-transcriptional level, whereas p53 in chemically transformed (Meth A) mouse cells seemed to be at the level of RNA translation [18,19]. p53 also participates in the transformation of normal embryonic cells and can cooperate with the activated Ha-ras oncogene to transform normal embryonic cells [20]. It showed a short-life of 5–10 min in non-transformed cells. In the developing fetus, a marked reduction of p53 mRNA levels was observed from day 11 onwards [21].

Earlier studies have suggested that p53 has a tumorigenic effect and can convert normal fibroblasts to tumorigenic cells e.g., [22]. However, further studies showed that the originally cloned p53 cDNAs used in the earlier studies contained dominant negative mutants within a conserved region of p53. These mutations enhanced its conformation and biological functions. The studies conducted in the late 1980s confirmed this notion and showed that the wild-type p53 acted as a tumor suppressor gene and inhibited oncogene transformation [23]. It was also shown that the p53 gene frequently mutated in human colorectal cancer. These findings, together with further work, showed that native p53 acted as a tumor suppressor gene. In fact, several studies have confirmed the established conclusion that genuine wild-type p53 act as a tumor suppressor gene and showed some characteristics of wild-type p53. Essential findings regarding p53 research in this area can be summarized as follows:

First, wild-type p53 can suppress transformation by the mutant p53 gene plus ras-activation [23]. Second, mutations in the p53 gene represent the most common alteration in genes in human tumors. Disruption of normal p53 function often leads to the inhibition and progression of cancer [24], and there are geographic variations of the p53 mutational profile [25]. Third, mutant p53 proteins may act as inhibitory proteins competing with and blocking the activity of wild-type p53 protein [26]. Modified p53 can also block MDM2-p53 binding, resulting in killing tumor cells overexpressing MDM2. As shown by Koom et al. [27], the combination strategy for the disruption of MDM2-p53 interaction with radiotherapy demonstrated enhanced antitumor effects in vitro and in vivo. p53 is a transcription factor, and it shows its tumor suppressive functions through the transcriptional regulation of genes [28]. In normal cells, the activity of p53 is shallow. p53 responds to a range of stress signals including DNA damage, oncogene activation, and hypoxia. Therefore, it results in the overexpression and accumulation of p53 in high concentrations upon stress signals. p53 then binds to a specific DNA sequence (termed the p53 responsive element (RE)) and hence transcriptionally regulates the expression of the genes involved. Studies have enabled the identification of the biological functions of the p53 gene including cell cycle control, apoptosis, senescence, DNA repair, change in metabolism, and metastasis [29,30]. These p53 functions can be modulated or inhibited by interaction with specific cellular oncoproteins such as MDM2. Details of such interactions are discussed in the next sections of this review. p53 protein concentration is mainly controlled by posttranscriptional modifications. These modifications include ubiquitination, phosphorylation, acetylation, methylation, and SUMOylation. Compared to other changes, ubiquitination is the major modification to regulate p53 [31] and the stability of p53 is regulated by diverse deubiquitinating enzymes [32].

The central controller of p53 is MDM2, which is a negative regulator of p53. In this process, MDM2 binds directly to p53 and mediates the ubiquitination-dependent degradation of p53. Fourth, recently, the MDM4 (MDMX) protein has been identified as a structural homology of MDM2, which binds directly to the p53 transactivation domain and inhibits its transcriptional activity. Therefore, MDM4 contributes to tumor function and progression. Interestingly, MDM4 can interact with the MDM2 protein via the RINg finger domain and inhibit degradation of the MDM2 protein, therefore regulating the role of MDM2 in inhibiting p53 activity. Thus, in different types of human cancers, both MDM2 and MDM4 are frequently overexpressed. In human HCC, it has been shown by Schlaeger et al. [33] that MDM4 and EFF1A2 can act as etiology-independent oncogenes in a significant percentage of HCC.

## 3. Role of p53 in Human Hepatocellular Carcinoma (HCC)

In HCC, p53 gene alteration is not uncommon, and there is a strong association between aflatoxin B1 exposure and the specific R249S mutation in nearly half of all cases and a higher percentage of these patients were Hepatitis B virus (HBV)-infected [34,35]. The down-regulation of the tumor suppressor protein p53 is linked to p53 gene mutation. The location, nature, and type of mutation depend upon the tissue origin of the cancer and carcinogens responsible for genetic alterations. A study from Taiwan showed that one of the most critical mutations among these mutations was a hotspot G to T mutation at amino acid position 249 [36]. The authors examined 20 different primary HCC samples (17 were hepatitis B surface antigen positive) using restriction fragment length polymorphism (RFLP) analysis. They found a specific loss of heterozygosity in only three out of the 20 samples examined. These three examples were found to carry a point mutation within the remaining p53 allele. None of these mutations were at the proposed aflatoxin hotspot of amino acid 249. The study showed that p53 mutations infrequently occurred in HBV-positive primary hepatomas in Taiwan (approximately 18%). The p53 mutation appears to be acquired later in tumor development [36].

Another study covering HCC in Southern Africa showed allelic deletions from chromosome 17 p and mutations of the p53 gene in 50% of primary HCC. Four out of five mutations detected (80%) were G.T substitutions with clustering at codon 249. This mutation could reflect exposure to aflatoxin B1, a contaminant in food in Africa, which caused the G to T substitution and is responsible for the development of HCC [37].

A study from India supported these findings. The study found a high frequency of HBV infection reaching more than 90% in HCC together with a low frequency of p53 mutation, which indicated that mutations in the p53 gene frequently found in HCC may not play a direct role in the development of HCC in India. The authors believe that HBV infection and possibly exposure to aflatoxin B1 (in food) appear to play a major role in the molecular pathogenesis of HCC in India [38].

Recently Ikeda et al. assumed that the molecular analysis of the HCC mutational landscape may provide more insights into the role of TP53. The authors conducted ctDNA next-generation sequencing (NGS) on 26 patients; 10 of these patients had tissue NGS. The authors demonstrated that 23 out of 26 patients had characterized alteration, and all these individuals had ≥1 potentially actionable alteration. The most frequently mutated gene was TP53 (16 out of 26 patients, 61.5%) and there were 47 unique characterized molecular alterations amongst 18 total gene alterations. The study showed that ctDNA profiling was feasible in HCC, and serial assessment using ctDNA NGS could reveal genomic changes with time [39].

Morishita and colleagues also used next-generation sequencing (NGS) to systematically identify gene mutations and reveal the pathogenesis of HCC. They found that the most common somatic mutations identified were tumor protein 53 (*TP53*; 35.6%) and β-catenin 1 (*CTNNB1*; 30.5%). They also found, at a low frequency in patients with HCC, other somatic mutations including those in genes encoding colony-stimulating factor 1 receptor (5.1%), epidermal growth factor receptor (6.8%), and others. The *TP53* mutations in tumors at advanced stages were significantly more frequent when compared with those in early-stage tumors. The results of the present study offer new insights and improved understanding of the etiology and the development of HCC [40]. Table 1 summarizes other studies related to p53 mutations and the use of NGS technique.

## 4. Murine Double Minute 2 (MDM2)-p53 Interactions in HCC

MDM2 (Murine Double Minute 2) has been shown to negatively control p53 functions by ubiquitination and degradation, and finally inhibition of p53 gene transcription and translation [49]. Factors that regulate ubiquitination of MDM2 and p53 may affect the cell fate and the ratio between MDM2 and p53 [50]. Several mechanisms have been identified to regulate MDM2 and p53 interactions [51]. These interactions may include:

### 4.1. Ribosomal Proteins

The activation of the MDM2-p53 regulatory loop by ribosomal proteins (RPs) plays a significant role in mediating nuclear stress and imposing cell cycle arrest and apoptosis [52]. Nuclear/ribosomal stress may result from the knockdown of nuclear proteins, which leads to the sequestration of MDM2 and inhibition of its ubiquitin ligase activity against p53 [53]. The disruption of ribosome biogenesis has been shown to cause p53 stabilization. The mechanism underlying this is related to the release from the nucleolus of several ribosomal proteins that bind to MDM2 and stop its inhibitory activity towards p53 [54]. Several subunit ribosome proteins rpL5, rpL11, and rpL23 have shown the ability to bind to MDM2, thus inducing p53 stabilization by inhibiting E3 ubiquitin ligase. Zhang et al. [55] reported that the human homolog of MDM2, HDM2 (the equivalent human protein), binds to ribosomal protein L11. The binding site is a central region in HDM2, which results in the prevention of HDM2-mediated p53 ubiquitination and degradation, and the restoration of p53-mediated transactivation and the accumulation of the p21 protein. The study supported the notion that L11 functions as a negative regulator of HDM2.

### 4.2. Ribosomal RNA

Ribosomal RNA is the RNA component of the ribosome, essential for protein synthesis, and comprises the dominant component within the ribosomes. A down-regulation of rRNA synthesis induced by silencing the RNA polymerase I subunit A (POLR1A) gene coding for the RNA polymerase I catalytic subunit was found to cause the stabilization of p53 without causing changes in nucleolar integrity in human cancer cells. The p53 stabilization was due to the inactivation of the MDM2-mediated p53 degradation. However, p53 stabilization did not occur when rRNA synthesis down-regulation was associated with a reduction of protein synthesis. The findings demonstrated that the balance of rRNA and ribosomal proteins synthesis controls the functions of p53 in mammalian cells [56].

### 4.3. MicroRNAs

MicroRNAs (miRs) are small non-coding RNA with regulatory functions involving the transcriptional and post-transcriptional regulation of hundreds of target genes. Usually, their expression is deregulated in cancer. In human cancers, there is a global decrease in micro-RNAs, indicating that they may play a role in tumor suppression. Therefore, microRNAs (miRs) can exert various biological functions by targeting oncogenes or tumor suppressive genes. The first microRNAs involved in the p-53 tumor suppressor network were reported in 2007 and they belong to the miR-34 family, these being miR-34a, miR-34b, and miR-34c [57]. The authors compared microRNA expression profile of wild-type and p-53-deficient cells and found that the expression of microRNA family members (miR-34a-c) reflected the p53 status and the genes encoding miR-34 family members were transcriptional targets of p53 in vivo and in vitro. Therefore, the p53 network suppressed tumor formation through a number of coordinated interactions and several transcriptional targets including the role played by miR-34 family members in inhibiting unregulated cell proliferation and tumor development [58,59].

The role of miR-125a in hepatocellular carcinoma was studied by Bi et al. [60]. The observed decrease in miR-125a expression in both HCC tissues and cell lines was associated with aggressive pathological features. The study indicated that miR-125a inhibited the proliferation and metastasis of HCC by targeting matrix metalloproteinase II (MMP II) and vascular endothelial growth factor A (VEGF-A) in vivo and in vitro. Therefore, miR-125a up-regulation may be a promising therapeutic agent in HCC.

Studies involving the role of miR-125b showed that it had a negative regulatory effect on p53 and p53-induced apoptosis in cells exposed to stress, indicating that it has a tumorigenesis effect e.g., [61].

One of the abundant miRs in the liver tissue is miR-122, which has been shown to act as a tumor suppressor agent in HCC. Decreased expression of miR-122 is frequently associated with poorly differentiated cancer, large tumors, metastases, and poor prognosis [62]. Recently, Simerzin and colleagues [63] showed that miR-122*, the passenger strand of miR-122, targeted MDM2 and participated as an important player in MDM2-p53 interaction. There was a significant negative correlation between the levels of miR-122* and MDM2 in human HCC samples. In vivo studies showed that miR-122* was capable of inhibiting tumor growth by emphasizing the tumor-suppressor characteristics of miR. By blocking miR-122 in murine livers with an antagomiR-122 (miR inhibitor), miR-122* accumulated, MDM2 was repressed, and then the p53 protein was elevated. The study showed that miR-122*, the passenger strand of miR-122, regulated the activity of p53 by targeting MDM2. Therefore, miR-122* has a tumor suppression activity which was previously attributed solely to miR-122. Further studies are needed to assess its effectiveness as a therapeutic agent in HCC and what key differences exist between miR-122* and miR-122.

Kim et al. [64] found that p53 up-regulated miR-200 and miR-192 family members and described the role of p53 in regulating epithelial-mesenchymal transition (EMT) in HCC through the induction of specific effector microRNAs including miR-141, miR-192, miR-194, miR-200b, miR-200c, and miR-215. These microRNAs targeted two of the principal EMT-activity transcription factors: the zinc-finger E-box-binding homebox 1 and 2 (ZEB1 and ZEB2). In human HCC and HCC cell lines, p53-upregulated microRNAs suppressed ZIB1 and ZEB2 genes, and as a result, EMT was suppressed resulting in increased E-cadherin and decreased vimentin expression.

### 4.4. LIM Domain Protein Enigma

The LIM domain protein (LIM domain refers to an evolutionary double zinc finger found in a variety of proteins) Enigma, PDLIM 7 (Enigma LIM) has been shown to interact with proteins involved in protein kinases, and the Enigma gene has been identified in human livers and stomach cancers. It has also been shown that the LIM domain protein Enigma directly interacts with MDM2 to form a complex with p53 in vitro and in human hepatoma cell lines and mouse embryonic fibroblasts. Additionally, it has been shown that the Enigma elicited p53 degradation by inhibiting MDM2 self-ubiquitination and increasing its ubiquitin ligase activity towards p53 [65].

### 4.5. Serum Response Factors

Phillips et al. [66] showed that in a well-characterized non-transformed human fibroblast model, serum growth factors were able to induce the expression of HDM2 with rapid kinetics. In their study, they explored the mechanistic response for these changes; the authors reported that the elevated HDM2 protein, which followed growth factor stimulation, was primarily the result of phosphatidylinositol-3 kinase-dependent stabilization of the HDM2 protein together with a global increase in protein synthesis. A recent study showed that increased Enigma transcription via the induction of serum response factor led to HDM2 stabilization and subsequent p53 degradation. The authors observed such changes in the livers of mice treated with hepatocyte growth factor (HGF). They also found that the serum response factor and Enigma in human liver and stomach cancers co-expressed with MDM2, but not p53. Furthermore, it was also found that Enigma promoted cell survival and induced chemoresistance by suppressing p53-mediated apoptosis of cell lines and a mouse xenograft model.

### 4.6. Phosphatase of Regenerating Liver 1

The phosphatase of the regenerating liver (PRL) family is a group of protein tyrosine phosphatase that has been implicated as oncogenic and found to show overexpression in a variety of cancers including colorectal [67], gastric and liver cancers, and particularly in metastatic cancers. Within this family, PRL-1, PRL-2, and PRL-3 showed a higher degree of structural sequence similarities. Min et al. [68] reported that overexpression of PRL-1 reduced the level of endogenous and exogenous p53 and inhibited p-53-mediated apoptosis. The down-regulation of p53 was mediated by p53 ubiquitination and subsequent proteasomal degradation. The ubiquitination of PRL-1 was achieved through two main mechanisms: (1) the induction of PIRH2 (p53-induced RING-H2) transcription, and (2) the induction of MDM2 phosphorylation through Akt signaling. These findings indicated that down-regulation of p53 through a negative feedback mechanism induced by PRL-1 contributed to cancer development. Further studies by the same group revealed that PRL-3 negatively regulated p53 in a pattern similar to PRL-1 via the activation of PIRH2 and MDM2 in cancer cells [69].

### 4.7. MDM2 Binding Protein

Earlier studies have shown that the MDM2 Binding Protein (MTBP) functioned as a metastasis suppressor. Furthermore it has been demonstrated that MTBP inhibited invasion regardless of the status of p53 [70]. Recently Bi et al. [1] demonstrated the metastasis suppressive function of MTBP in HCC and reported the reduced expression of MTBP in approximately 70% of human HCC cells when compared to adjacent non-tumor tissue. This reduction was also associated with the local invasion of lymph node metastasis. The expression levels of MTBP were negatively correlated with a migratory potential of HCC cells regardless of p53 status. These results were consistent with the work of Vlatkovic et al. [71] who demonstrated that reduced MTBP was associated with poor prognosis in head and neck cancers. However, several gaps are still unresolved in this area. For example, it is still not clearly understood how MTBP expression is regulated in HCC cells. In addition, the functional correlation between MTBP and MDM2 is not clearly understood. In the meantime, we need to know why these changes are not p53 dependent.

Another factor that may also affect the interaction of p53 with MDM2 is the presence of a single nucleotide polymorphism (SNP309) in the MDM2 promoter. This factor has been shown to increase the affinity of the transcriptional activator Sp1, and as a result, provides higher levels of MDM2 RNA and protein plus the subsequent attenuation of the p53 pathway. Such processes have been shown to increase tumor formation in humans, hence the proposal made by the authors to use SNP309 as a rate-limiting event in carcinogenesis [72]. Table 2 summarizes the key studies showing the role of p53 and MDM2 in HCC development.

Although there have been several trials on new therapeutic approaches in human hepatocellular carcinoma, we are yet to reach a definite recommended drug therapy. Nutlin-3 has significant anticancer effects against human HCC cells, regardless of p53 status [86]. Recently, Nutlin-3 has shown the ability to regulate tumor cell migration, invasion, metastasis, and drug resistance, and it has demonstrated the ability to reverse the epithelial-mesenchymal transition (EMT) in gemcitabine-resistant HCC cells [87]. Studies have also shown that p53-MDM2 binding antagonists such as Nutlin-3 usually select p53 mutations present at a low frequency at diagnosis, leading to resistance, but such tumors may nevertheless remain responsive to irradiation therapy [88]. It is important to note here that p53 plays a role in the radiotherapy response of HCC [89].

## 5. Effects of Nutlin in HCC

In 2004, Vassilev and colleagues [90] were the first to report the identification of a small and potent molecule, Nutlin, which acts as an antagonist of MDM2. Nutlins-1, -2, and -3 showed potency against MDM2-p53 binding. They inhibit the MDM2-p53 axis by mimicking the interaction of critical amino acid residues with the hydrophobic activity of MDM2. By binding with MDM2 in the p53-binding pocket, Nutlin activates the p53 pathway in cancer cells, resulting in cell cycle arrest, apoptosis, and the growth inhibition of human tumor xenografts in nude mice [90]. The effects of nutlins have been evaluated in hematological cancers [91,92], breast cancer [93], human medulloblastoma cells [94], ovarian cancer [95], nasopharyngeal carcinoma [96], and esophageal squamous cancer [97]. 

Recently, the mechanism by which Nutlin-3 induces antitumor activity in HCC cells was studied by Wu and colleagues. The study elucidated the role of Nutlin-3 in reversing the epithelial-mesenchymal transition in gemcitabine-resistant HCC cells. The authors investigated the effect of Nutlin-3 treatment on cell growth, migration, and invasion of both parental HCC cells and gemcitabine-resistant (GR) HCC cells. The work showed that Nutlin-3 inhibited cell migration and invasion in the GR HCC cells. The authors concluded that Nutlin-3 played a role in reversing epithelial-mesenchymal transition in GR cells through the regulation of Smad2 (transforming growth factor-β) expression, suggesting that Nutlin-3 could offer a potential therapeutic agent in treating HCC, particularly in the presence of gemcitabine resistance [87].

Nutlin-3 also causes the stabilization and accumulation of p53 in cells, leading to the activation of target genes (e.g., p21) and MDM2. This effect was claimed to be dependent on the presence of wild-type p53 as cells where p53 is deleted do not respond to Nutlin treatment [90]. However, the work by Wang and colleagues [74] showed that regardless of p53 status, Nutlin-3 had a significant anticancer effect against human HCC. They also revealed that Nutlin-3 treatment increased apoptosis in three human HCC lines with wild-type (HepG2), mutant (Hep3B), and null p53 (Huh7) as well as increased expression of Bax, Noxa, and PUMA. The effectiveness of Nutlin-3 in treating HCC has also been demonstrated by Zheng et al. [98]. They also showed the ability of Nutlin-3 in Doxorubicin-resistant HCC.

Although arsenic trioxide has demonstrated effective anticancer actions against leukemia and solid tumors, poor effects were observed when used for treating HCC, which might be due to drug resistance. Zheng and colleagues [99] showed that acquisitions of p53 mutations contributed to the resistance of HCC to arsenic trioxide. They also demonstrated that Nutlin-3 could overcome arsenic trioxide resistance and inhibit HCC tumor metastasis through p73 activation and promote mutant p53 degradation mediated by arsenic trioxide.

The study by Shi et al. [100] added to our understanding of the mechanisms by which Nutlin-3 works in HCC cells. Their work was based on previous knowledge that the reactivation of p53 was an attractive therapeutic strategy in HCC, particularly with disrupted-p53 functions. The authors investigated the relationship between Nutlin-3 and p53 phosphorylation on serine 392 protein expression level in SMMC-7721 (wild-type TP53) and HuH-7 cells (mutant TP53). Their work showed that Nutlin-3 induced apoptosis in HCC cells through the down-regulation phosphor-Ser392-p53. They concluded that inhibition of p53 phosphorylation on serine 392 could be another therapeutic approach in HCC.

There is evidence that the binding of p53 follows the disruption of the interaction between p53 and MDM2 in the presence of Nutlin to other C-terminal modifiers such as interferon gamma inducible protein 16 (IFI16) [101]. IFI16 belongs to the PYHIN family, which contains a pyrin domain (PYD) at the N-terminus and two C-terminal HIN-200 domains (HIN refers to Hematopoietic expression, Interferon-inducible, and Nuclear localization domains), HIN-A and HIN-B, which can secure double strand DNA (dsDNA) [101]. In this context, IFI16 is a DNA sensor in the cellular nucleus, and it modulates the function of p53 and inhibits cell growth. Shi et al. [102] showed that Nutlin-3 regulated the subcellular localization of IFI16 in vivo in a p53-dependent manner. On the other hand, the loss of IFI16 caused deregulation of p53-mediated apoptosis leading to cancer development [103].

The work of Giovannini et al. [104,105] showed that the Notch3 receptor silencing in HCC resulted in p53 up-regulation in vitro; they followed this work further and aimed to study the mechanisms that associated Notch3 receptors to p53 protein expression. They explored the regulation of p53 by Notch3 signaling in three HCC cell lines: HepG2, SNU398, and Hep3B. They found that Notch3 regulated p53 at the post-transcriptional level, controlling both CyclinG1 expression and the feed-forward circuit involving p53, miR-221, and MDM2. The findings were validated in human HCC and a rate model for HCC. The results indicated the inactivation of the Notch3 receptor as a novel therapeutic approach for increasing drug sensitivity. The work also showed that Notch3 silencing enhanced the effects of Nutilin-3 in HCC and hence the applicability of this research in managing HCC [106].

## 6. Future Directions and Therapeutic Applications

With the wealth of findings outlined in this review, one might wonder about the future research directions, particularly at these three levels: (1) biomarkers that can help in the early detection of HCC or recurrence and are based on the MDM2-p53 interaction; (2) studies that correlate tumor characteristics including MDM2-p53 and survival pattern of patients with HCC; and (3) therapeutic interventions that are directed at MDM2 inhibition, thereby restoring p53 function or stimulating p53 functions. With these three research levels in mind, one may raise the following research questions/directions:

First, MDM2-p53 as biomarkers of HCC. The recent study by García-Fernández and coworkers showed that it was possible to detect the mutated p53 gene with cirDNA and that it could be possibly used as a biomarker of tumor recurrence during the evolution of the transplanted patients [107]. Much work is needed in this area. The identification of sensitive and specific biomarkers may offer new approaches for intervention at the early stages of HCC development/recurrence.

Second, MDM2-p53 and prognosis of patient survival. Several studies have highlighted the use of MDM2-p53 interaction as a prognostic indicator. Endo et al. [108] showed that MDM2 was expressed in 26% of HCC and its expression correlated positively with p53 mutations. They recommended MDM2 overexpression as a useful predictor of poor prognosis in patients with HCC following hepatic resection. Zhang et al. [109] found that p53, p21/WAF1, and MDM2 proteins were overexpressed in all HCC cases included in their study and that there was a positive correlation between p53 and p21/WAF1 overexpression. They also found that the expression of p21/WAF1 and MDM2 could be considered as useful prognostic indicators in patients with HCC. Recently, the work of Yang et al. [81] showed that the combined p53 Pro/Pro and MDM2 G/G genotype increased the risk of developing HCC and could be used as an independent adverse prognostic indicator in the early stages of HCC. However, further studies are needed in this area to assess the predictive validity of these prognostic indicators and the practicality of their use in clinical practice.

Third, MDM2-p53 and new therapeutic approaches. This review outlined the role of p53 in killing tumor cells. Therefore, strategies to restore mutant p53 gene function or activate p53 could provide new approaches in cancer therapy. Considering the central role of MDM2 in modulating p53 function, much attention should be directed to developing MDM2 inhibitors that can restore p53 function or stimulate these functions. As discussed earlier, such approaches for HCC therapy have been demonstrated in Nutlin-3, a small molecule inhibiting MDM2-p53 interaction and causing p53 activation. Nutlin induced its action through certain mechanisms, but the central mechanism for its effect is to mimic the MDM2-binding p53 peptide that inhibits MDM2-p53 interaction [90]. Some approaches are promising in this area; they follow the mechanisms related to the inhibition of MDM2-p53 interaction. Interestingly, several compounds are currently entering phase I trials, and have shown the ability to antagonize MDM2 function. These compounds include NVP-CGM097 [110] and MK-8242 [111]. The studies conducted on SIRT1 deacetylation showed its ability to maintain the deacetylated status of the p53 protein, thus promoting its MDM2-mediated degradation. Other studies have shown the role of SIRT1 in the biology of liver tumors containing cancer stem cells [112]. Tenovins, as it is the case with other SIRT1 and SIRT2 inhibitors, have presented promising results showing stabilization and hence activation of p53 [113]. These new approaches look promising and could provide future therapy to patients with HCC.

Further research is needed to explore the p53-dependent cancer cell death pathways targeting MDM2. Agents that are capable of disrupting MDM2-p53 interaction with the resultant increase of p53 could provide a sound therapeutic approach. Furthermore, targeting overexpressed MDM2 on cancer cell membranes could also cause membrane destabilization and rapid cancer-specific necrosis [114].

## 7. Conclusions

The knowledge that has accumulated since 1979 on p53 and MDM2 and their roles in the development of cancer mainly HCC is rapidly growing. This review focused on the primary regulatory mechanisms of MDM2 in inhibiting p53 functions and the biological factors affecting such interaction. However, despite this wealth of knowledge, this article raised some questions and identified gaps in the research that await answers. Which of the biological factors examined in this study have the dominant role in cancer development? How do such interactions affect biological processes such as cellular apoptosis? How can we interfere at an earlier stage to slow or stop the cancer development process? With these questions in mind, the implications of current knowledge and future research should focus on three levels: the role of early detection in HCC or recurrence, their use as prognostic indicators, and the development of new therapeutic interventions. The studies related to Nutlin-3 in HCC present a model for clustering new therapeutic interventions that could follow the mechanisms outlined concerning MDM2-p53 interplay. However, more research regarding the biology of MDM2-p53 interaction may offer a deeper understanding of the mechanisms and provide more effective therapeutic interventions for patients with HCC.

## Figures and Tables

**Table 1:** Summarizes the role of p53 mutation in human hepatocellular carcinoma (HCC) in light of the recent studies using next generation sequencing (NGS) technique.

Author [Reference]	Study Design/Study Purpose	What Was Exained?	Key Findings	Conclusions
Lu et al. [41]	Cancerous and non-cancerous specimens from 12 patients with HCC from western China.	A 372 cancer-associated genes including TP53 were screened using NGS technique.	Results confirmed mutations of previously identified HCC drivers including p53 and Kras.	Additionally, mutations in several cancer genes which had not been previously associated with HCC were identified including RUNX1 and JAK 3.
Huang et al. [42]	Exome sequencing to identify somatic mutations.	The whole exome (10 samples) and validation set (100 samples), in HBV positive with HCC.	*TP53* was the candidate driver in 27% (it was the most frequently mutated tumor suppressor).	The study provided a view of the somatic mutations that may be implicated in advanced HCC.
Kan et al. [43]	Identify genetically altered genes and pathways implicated in HBV-associated HCC.	A whole-genome sequencing study of 88 matched HCC tumor/normal pairs, 81 of which were Hepatitis B virus (HBV) positive.	*TP53* was the most frequently mutated tumour suppressor (35.2%). Beta-catenin is the most frequently mutated oncogene (15.9%).	The study also identified several prevalent and potentially actionable mutations, including activating mutations of Janus kinase 1 (JAK1), in 9.1% of patients.
Ahn et al. [44]	Exome sequencing to identify somatic mutations.	A whole exome sequencing and copy number analysis was performed on 231 hepatocellular carcinomas (72% with hepatitis B viral infection) that were classified as early-stage hepatocellular carcinomas.	Recurrent somatic mutations were identified in nine genes, including TP53, CTNNB1, AXIN1, RPS6KA3, and RB1.	RB1 mutations can be used as a prognostic molecular biomarker for resectable hepatocellular carcinoma. However, further studies are needed to explore other roles played by mutations.
Totoki et al. [45]	Use of NGS techniques in understanding the genetic changes in HCC genomes.	A collection of data from 503 liver cancer genomes uncovered 30 candidate driver genes and 11 core pathway modules.	*TP53* was the candidate drive in 31% of cases.	De-regulation was detected in p53 signaling (72%), Wnt signaling (66%), Chromatin remodeling (67%), Telomere maintenance (68%). Newly identified alterations in genes encoding metabolic enzymes, chromatin remodelers and a high proportion of mTOR pathway activations offer potential therapeutic and diagnostic opportunities.
Schulze et al. [46]	Exome sequencing to identify somatic mutations.	Exome sequencing analysis of 243 liver tumors identified mutational signatures associated with specific risk factors; combined alcohol and tobacco consumption and exposure to aflatoxin B1.	The authors identified 161 putative driver genes associated with 11 recurrently altered pathways. Associations of mutations defined 3 groups of genes related to risk factors: *CTNNB1* (alcohol), *TP53* (hepatitis B virus) and *AXIN1.*	The study identified risk factor-specific mutational signatures and defined the landscape of altered genes and pathways in HCC.
Niu et al. [47]	An update on recurrently mutated genes including p53 and deregulated signaling pathways in HCC using NGS techniques.	Current literature	*TP53* mutation has been identified as the most frequently molecular alterations in HCC and can be used to predict HCC development and is associated with shorter survival time.	Understanding genetic alterations in HCC could provide new insight into newly targeted therapies.
Schulze et al. [48]	A review on genetic profiling of hepatocellular carcinoma using NGS	Current literature	Differences in mutation rates of cancer drivers and associated pathway among different studies may be partially due to clinical heterogeneity. In hepatitis B virus –related HCC, inactivating mutations of *TP53* and *KMT2B* are more frequently involved.	NGS data will soon allow to better understanding of tumour heterogeneity and its potential role in treatment decision-making.

**Table 2:** Role of p53 and murine double minute 2 (MDM2) pathways in hepatocellular carcinoma development.

Author, [Reference]	Study Design	What Was Examined?	Key Findings	Conclusions
Higashitsuji et al. [73]	In vivo and in vitro	The effect of Gankyrin on p53 and MDM2 interactions.	Gankyrin, an ankyrin repeat oncoprotein overexpressed in HCC, enhances MDM2 autoubiquitination in the absence of p53.	Gankyrin is a cofactor that increases the activities of MDM2 on p53.
Jalbkowski et al. [74]	Specimens from patients with HCC, and patients with cirrhosis linked to HBV infection.	The expression of p53 and MDM2.	Mutation of p53 gene and MDM2 immunopositivity were detected more in HCC. MDM2 positivity was not associated with MDM2 amplification .	Besides p53 alteration, MDM2 gene deregulation plays a role in HCC.
Yoon et al. [75]	Samples from patients with HBV with and without HCC	Evaluation of the association of MDM2 and p53 polymorphisms with the presence and onset of HCC in Korean patients with HBV infection.	Multivariate analysis for the presence of HCC revealed an odd ratio for MDM2 G/G over T/T of 4.89 and for p53 Pro/Pro over Arg/Arg of 3.03. Both were significant.	MDM2 and p53 are associated with early development of HCC in Korean patients with HBV infection.
Edamoto et al. [76]	Samples from patients with HCV infection, HBV infection, and excessive alcohol intake.	Screening the three groups for alterations in genes involved in RB1 pathway, p53 pathway and Wnt pathway.	Alterations in the p53 pathway consisted mostly of p53 mutations or p14 promoter methylation. Mutations of the p53 gene were found in similar frequency.	RB1, p53, and Wnt pathways were commonly affected in HCC of the three groups.
Lim et al. [77]	Human hepatoma cells, and human hepatoblastoma cells.	The relationship between p53 and Snail proteins in HCC and whether Snail and p53 contribute to HCC.	Only p53 wild-type induced endogenous Snail protein degradation via MDM2 ubiquitination whereas p53 mutant did not induce Snail degradation.	The results show the role of p53 mutation and Snail overexpression as a late event in HCC development.
Qiu [78]	Samples from patients with HCC	The relationship between MDM2 gene expression and p53 gene mutation in HCC and their correlation with the invasiveness of the disease.	MDM2 genes overexpression was found in 26% of samples while p53 mutation was found in 47% of samples.	Either MDM2 overexpression or p53 mutation may be related to the invasion of HCC.
Xian et al. [79]	Human hepatoma cells	Identify the underlying mechanism by which p53 induce hepatitis B virus X protein (HBx) degradation and express its oncogenic functions.	Over expression of p53 protein reduces the level of HBx protein and shortens its half-life. In MDM2 knock out cells, p53 can accelerate turnover of HBx protein.	P53 mediated HBx degradation in MDM2-dependent. MDM2 interacts with HBx but does not promote its ubiquitination. In HCC tissues with wild-type p53, HBx protein is hardly detected.
Kim et al. [64]	Tissue specimens from HCC patients	Test the role of epithelial-mesenchymal transition (EMT) in tumor progression and metastasis and the role of transcription factor ZEB1 and ZEB2 in regard to HCC.	P53 up-regulates microRNA including miR-200 and miR-192 family members. Inhibition of miRNAs affects p53-regulated EMT by altering ZEB1 and ZEB2 expression.	P53 can regulate EMT in HCC.
Eferl et al. [80]	Mice carrying a “floxed” c-Jun allele.	Study the effect of liver-specific inactivation of C-Jun at different stages of tumor development and its role in development of HCC in mice.	The number and size of hepatic tumors was dramatically reduced when c-Jun was inactivated after the tumor was initiated. The impaired tumor development was associated with increased levels of p53.	c-Jun prevents apoptosis by antagonizing p53 activity. This may illustrate a possible mechanism in early stages of HCC development.
Yang et al. [81]	Patients with HCC, non-HCC patients and control	Investigate the influence of combined p53 Arg72 Pro and MDM2 SNP309 on the risk of developing HCC in patients with chronic hepatitis B virus infection.	Patients with combined p53 Pro/Pro and MDM2 G/G genotypes had a poorer prognosis than other genotypes.	There is increased risk for HCC in the presence of p53 Pro/Pro and MDM2 G/G genotype in combination.
Dongiovanni et al. [82]	Immortalized mouse hepatocytes and rat livers.	Identify iron regulated gene pathways involved in HCC.	Iron down-regulated whereas deferoxamine up-regulated mRNA levels of MDM2, the ubiquitin ligase involved in the degradation of p53. Iron status affected p53 ubiquitination and degradation rate.	Iron status influences p53 activity and antioxidant response by modulating MDM2 expression.
Umemura et al. [83]	Specimens of HCC.	Assess the clinical significance of gankyrin overexpression in HCC by using monoclonal-anti-gankyrin antibody and immune-histochemical technique.	The cumulative survival rate of patients with gankyrin-positive HCC was significantly higher than that with gankyrin-negative HCC.	Gankyrin plays an important oncogenic role mainly in early stages of HCC.
Di Vuolo et al. [84]	Patients with viral hepatitis-related HCC and control.	Analyze the role of polymorphisms in the development of HCC.	Frequency of TP53 codon 72 alleles was not significantly different between cases and control. A significant increase of MDM2 SNP309 G/G and T/G genotypes were observed among HCC cases.	There is a significant role for MDM2 SNP309 G allele as a susceptibility gene for the development of viral hepatitis-related HCC.
Xie et al. [85]	Human hepatoma cells.	Identify the mechanism by which nucleolar protein, CSIG, regulates the MDM2-p53 pathway.	CSIG translocates from nucleolus to nucleoplasm in response to nucleolar stress. Knockdown of CSIG attenuates the induction of p53 and abrogates G1 phase arrest in response to nucleolar stress. CSIG interacts directly with MDM2 and inhibits MDM2 E3 ubiquitin.	CSIG-MDM2-p53 regulatory pathway an important role in cellular response to nucleolar stress.

HBV = Hepatitis B virus, HCV = Hepatitis C virus, CSIG = Cellular Senescence-Induced Gene.
